# Misunderstanding of Front-Of-Package Nutrition Information on US Food Products

**DOI:** 10.1371/journal.pone.0125306

**Published:** 2015-04-29

**Authors:** Lisa M. Soederberg Miller, Diana L. Cassady, Laurel A. Beckett, Elizabeth A. Applegate, Machelle D. Wilson, Tanja N. Gibson, Kathleen Ellwood

**Affiliations:** 1 Department of Human Ecology, University of California, Davis, One Shields Avenue, Davis, California 95616, United States of America; 2 Department of Public Health Sciences, University of California, Davis, One Shields Avenue Davis, California 95616, United States of America; 3 Nutrition Department, University of California, Davis, One Shields Avenue, Davis, California 95616, United States of America; 4 College of Southern Maryland, La Plata, Maryland 20646, United States of America; Duke University Medical Center, UNITED STATES

## Abstract

Front-of-package nutrition symbols (FOPs) are presumably readily noticeable and require minimal prior nutrition knowledge to use. Although there is evidence to support this notion, few studies have focused on Facts Up Front type symbols which are used in the US. Participants with varying levels of prior knowledge were asked to view two products and decide which was more healthful. FOPs on packages were manipulated so that one product was more healthful, allowing us to assess accuracy. Attention to nutrition information was assessed via eye tracking to determine what if any FOP information was used to make their decisions. Results showed that accuracy was *below* chance on half of the comparisons despite consulting FOPs. Negative correlations between attention to calories, fat, and sodium and accuracy indicated that consumers over-relied on these nutrients. Although relatively little attention was allocated to fiber and sugar, associations between attention and accuracy were positive. Attention to vitamin D showed no association to accuracy, indicating confusion surrounding what constitutes a meaningful change across products. Greater nutrition knowledge was associated with greater accuracy, even when less attention was paid. Individuals, particularly those with less knowledge, are misled by calorie, sodium, and fat information on FOPs.

## Introduction

**Fig 1:**
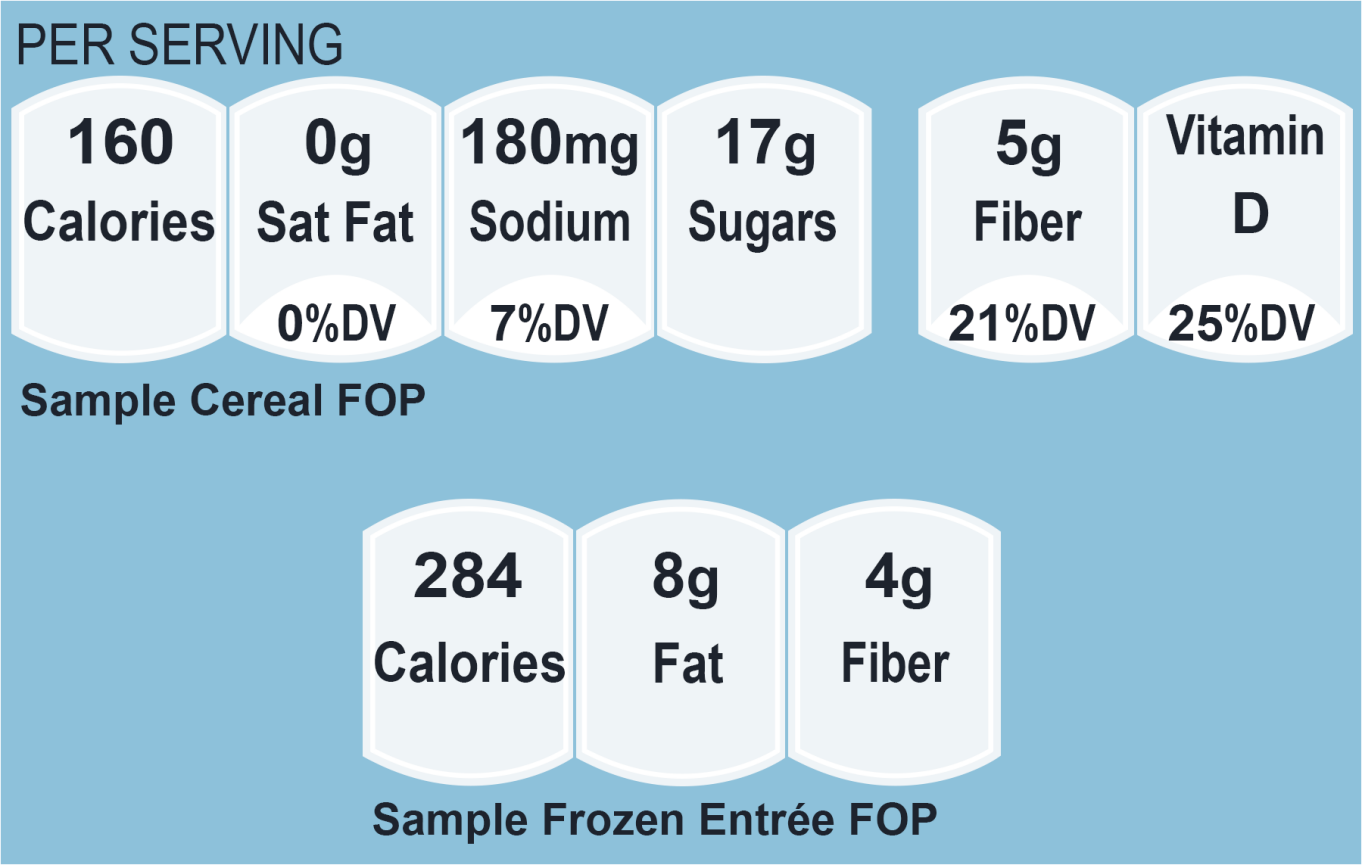
Front-of-Package (FOP) symbols for cereals (top) and frozen entrées (bottom).

Recent research indicates that consumers rely equally on healthfulness and taste when choosing foods to buy [1]. This could indicate a greater potential for individuals to take advantage of front-of-package nutrition symbols (FOPs), which have appeared more frequently on food packages over the last 10 years [2]. FOPs summarize key nutritional aspects of packaged foods based on information in the Nutrition Facts panel (NFP) including amounts, and where available, percent daily value (%DV) per serving. Although there are a variety of systems [2], FOPs in the US typically include calories, %DV for vitamins and minerals, and weight plus %DV for a small set of nutrients (see Fig 1 for examples). Unlike the more detailed Nutrition Facts panels appearing on the back of food packages, FOPs are not required on packaged foods in the US nor is the format regulated. Moreover, FOPs do not attempt to convey the specific recommendations of the USDA’s Dietary Guidelines for Americans to the same extent as do NFPs.

The Institute of Medicine (IOM) stated that FOPs could encourage more healthful food choices and contribute to the reversal of the obesity crisis. Their expert panel identified the potential benefits of FOPs, including making nutrition information easier to notice, understand, and use; educating consumers about the products they buy; and encouraging manufacturers to offer more healthful products [2]. Thus, FOPs should in theory be readily noticeable, place minimal demands on the individual, and require little prior nutrition knowledge to understand.

Many studies investigating FOP use have focused on the Traffic Light system [3–5] (uses colors and/or words to indicate whether levels of 3 or 4 nutrients are high, medium, or low), which is not typically found in the US. Research conducted in Europe shows that consumers prefer simplified information on the front of the package to the more complex nutrition table on the back [6], and that they make decisions more quickly, when simpler FOP nutrition information is presented [7, 8]. Some work also shows that consumers can use FOPs to select more healthful foods [9–13]. For example, Grunert [9] presented participants with pairs of FOPs and found that roughly 84% of participants were able to select the most healthful label, defined as the one with less fat, saturated fat, and calories (they were equal on sugar and salt). Other studies have also reported that consumers were able to understand FOPs [10], even under time constraints [12].

On the other hand, a recent online study conducted in the US showed that participants performed relatively poorly when using a star plus calorie FOP system in which products receive fewer stars if they exceeded the recommended values on nutrients that should be limited [14]. However, this system is not widely used in the US so it is possible that lack of familiarity could be contributing to the confusion. An FOP system, called Facts Up Front, is more commonly found in the US. This is a voluntary system promoted by the Grocery Manufacturers Association and the Food Marketing Institute. An industry-funded study used a large online US sample to examine use of Facts Up Front labels across an increasing amount of Facts Up Front information (number of cells) on package fronts [11]. Researchers compared four label conditions: no labels, just calories, adding nutrients to limit (with %DV), and adding nutrients to encourage. Although participants’ healthfulness accuracy was very high overall (87% to 96%), data showed that accuracy increased with increasing amounts of information in the FOP. Independently-funded research has also examined use of Facts Up Front labels. Within two studies, Bialkova and colleagues [15] used eye tracking to show that attention mediates the effects of nutrition labels on yogurt choice among university students in the UK. The researchers crossed two fat levels with three FOP styles, including a monochrome and a colorful Facts up Front style. In general, all FOP styles were able to communicate fat levels; however, the colorful style received the most attention. In another study, researchers assigned participants from an online panel to one of five label conditions: no label, two styles of Facts up Front, and two versions of Traffic Light symbols [13]. Participants compared two food products, and evaluated single products, in response to questions on specific nutrient levels. Although performance was high (80% accuracy) across FOP types, participant ratings indicated that the Facts up Front symbols were the most confusing. The work described above is important in showing that FOP style influences attention and accuracy of interpretation. However, in both cases, consumers were required to evaluate only one nutrient at a time, which may influence the generalizability of attention [15] and accuracy data [13, 15].

In its report, the Institute of Medicine suggested that prior nutrition knowledge should be unnecessary when using FOPs [2], yet only a handful of studies have examined how knowledge relates to comprehension and decision making accuracy. In a study conducted in the UK, researchers showed that nutrition knowledge was related to accuracy of healthfulness decisions but that interest in healthy eating was related to purchasing decisions [9]. On the other hand, Mejean reported that simple FOPs (e.g., tick marks and logos) may be particularly useful to those who have low levels of nutrition knowledge [16]. No measures of attention were included in these studies. In general, we know little about the relationship between nutrition knowledge and 1) attention to FOPs on food packages, and 2) accurate use of FOPs for food choice.

Thus, the literature remains unclear regarding 1) how well individuals in the US use Facts Up Front to select the more healthful foods, 2) whether prior knowledge supports accurate use of FOPs, and 3) and whether some nutrients in the FOP are more confusing than others. If accuracy is low, it is important to determine whether this is because some or all nutrients are not receiving attention or attention is failing to result in comprehension.

In the present study, we used eye-tracking methodology to investigate attention allocated to nutrition information presented in FOPs. Participants compared two similar foods, either two cereals or two frozen entrées, that differed in the information provided by the FOP so that one product was healthier. The healthier option was in line with the USDA/HHS Dietary Guidelines for Americans 2010 [17] recommendations to consume products with less sugar, sodium, or calories, or more fiber (described in greater detail below). We assessed the relationship of accuracy of the healthfulness choice to prior nutrition knowledge and to attention, measured as dwell time for the individual nutrients in the FOPs, and examined whether knowledge was associated with more attention and thus greater accuracy.

## Methods

We used stratified cluster sampling to recruit a socioeconomically diverse sample. Individuals were contacted by phone (n = 1286) and 630 were interested in participating. Of these, 238 people were excluded because they had experienced a neurodegenerative disease, head trauma, stroke, or rarely or never buy groceries for their household. Of the 392 individuals who met the study criteria, 5 dropped out prior to completing the study and 42 had poor quality eye tracking, leaving 345 participants in the final sample with eye tracking data and 387 participants with accuracy data. The Institutional Review Board of the University of California, Davis, approved the study and free and informed written consent of participants was obtained.

Participants viewed high-quality images of package fronts from two categories, cereals and frozen entrées, on a computer screen. All packages contained FOPs similar to those in Fig 1. The cereal FOPs matched the Facts up Front style and the entrée FOPs matched a similar style that is more commonly found on frozen entrees in the US. The FOPs on cereals contained percent daily values (%DV) and/or amounts (grams or milligrams) per serving of saturated fat, sodium, sugar, fiber, and vitamin D as well as calories per serving. FOPs for frozen dinners contained cells for calories, fat, and fiber.

To assess the extent to which individuals can accurately use FOPs to compare nutritional qualities of foods, we created 12 pairs of cereals and 12 pairs of frozen entrées, and manipulated the nutrition information so that one member of the pair was the more healthful choice (and pairs were counterbalanced on product-healthfulness). For each pair, we manipulated 1) one nutrient a great deal, referred to as the target nutrient, and 2) all other nutrients, referred to as the non-targeted nutrients, a trivial amount, in the opposite healthfulness direction. Sugar and fiber were targeted nutrients on cereals, and calories and fiber were targeted nutrients on frozen entrées. Targeted nutrients changed on average 100% in one direction (e.g., 8 versus 16 grams of sugar), whereas non-targeted nutrients changed on average 5% in the other direction (157 versus 151 milligrams of sodium). Thus, targeted nutrients carried the most salient healthfulness information for the comparison and “correct” choices were defined as the food with one of the following: 1) a great deal less sodium, 2) far fewer calories, or 3) a great deal more fiber, relative to the other option. This approach allowed us to examine the effects of specific nutrients on consumer attention within individuals, unlike FOP manipulations that present nutrients unsystematically [18, 19] and to relate specific nutrients to healthfulness accuracy. As a manipulation check, we compared the nutrient density scores, which represent another approach to defining relative healthfulness (NRF9.3; [20]), of pairs of products and confirmed that the more healthful product in each pair had a higher nutrient density score, paired t-test, p < .001. Individuals were shown a pair of products on a single screen and were asked to identify the more healthful choice by clicking on a button below the product of their choice. Random guessing would lead to 50% accuracy; basing choice on the target nutrient with greater difference would lead to higher accuracy, while distraction by the minor difference would lead to lower accuracy.

To determine the extent to which individuals paid attention to the various nutrients in the FOPs, eye movements were measured using a desk-mounted video-based eye tracker. A region of interest was defined for each FOP cell and the eye tracker measured dwell times for each region of interest. The EyeLink 1000 has a sampling rate of 1000 Hz and average accuracy of 0.25–0.5 degree. Pairs of items were presented on the computer screen, while eye movements were recorded; the comparison ended when participants selected their choice by a mouse click.

We assessed prior nutrition knowledge through a 25-item multiple-choice measure covering nutrition and health, nutrition facts, and procedural knowledge (e.g., what to look for when shopping for cereal) [21]. The score was the proportion of correct responses.

### Statistical Analyses

Our primary analytic question was whether there was an association between accuracy (proportion of trials for which participant correctly selected the healthier option) and either attention or nutritional knowledge. Attention was measured for each package pair as the proportion of total trial time spent in each FOP region of interest (3 for frozen entrées, 6 for cereals) and summarized descriptively. Knowledge was the proportion correct on the 25-item test. We first used the one-sample t test to test whether average accuracy was significantly above or below chance (50%) and the paired t test to test for significant differences between food types in the overall rate of accuracy above chance. A mixed effects ANOVA was used to test for differences in attention between the FOP types for each product, using the Tukey-Kramer correction for multiple comparisons. General linear regression models were used, separately for the cereal and entrée choices, to assess the two key hypotheses, that accuracy was associated with attention and with nutrition knowledge. All models were controlled for sex, age, education, household income, and BMI, to reduce unexplained variance and possible bias, and to assess whether specific demographic groups make less accurate use of FOP information. All analyses were performed using SAS software version 9.3. Model assumptions were validated using graphical examination of the model residuals, and possible collinearity of predictors was assessed by examining correlations.

## Results

Participants were 60% women, 74% white, and 47% single; 74% were parents (Table 1). They averaged 50 years of age, 16 years of formal education, and BMI of 28.1, with 37% overweight and 29% obese. The largest proportion, 19%, of participants, reported yearly household income between $50,000–$74,999. Participants averaged 16.5 (out of 25) on the nutrition knowledge measure with standard deviation (SD) 3.4. See Table 1.

**Table 1:** Characteristics of Study Participants.

Variable	N	Mean (SD) or percent %
*Percent dwell time*, *cereals*	345	35.6 (9.1)
*Percent dwell time*, *entrées*	345	27.5 (7.9)
*Accuracy*, *cereals*	387	38.2 (28.2)
*Accuracy*, *entrées*	387	63.1 (19.4)
*Age*	392	49.9 (16.5)
*Education* (years)	392	15.8 (2.5)
*Nutrition Knowledge*	392	16.5 (3.4)
*Sex* (Female)	392	60.0%
*BMI*	390	28.1 (6.4)
*Race*	390	
White		74.0%
African American		9.7%
Other		16.3%
*Income*	389	
Less than $10,000		4.9%
$10,000 to $14,999		6.4%
$15,000 to $24,999		6.2%
$25,000 to $34,999		8.7%
$35,000 to $49,999		14.4%
$50,000 to $74,999		19.3%
$75,000 to $99,999		18.0%
$100,000 to $149,999		15.2%
$150,000 to $199,999		4.6%
$200,000 or more		2.3%

Residual diagnostic plots for the mixed effects ANOVAs showed some skew, but given the sample size, mild deviations from the normal assumption are no strong cause for concern. Residuals for the general linear models showed excellent fit, and predictors had little collinearity.

Participants paid substantial attention to FOPs, spending 35.6% of total dwell time on FOPs for cereal (SD 9.1%), and 27.5% for entrées (SD 7.9%, Table 1). The proportion of time spent on each nutrient varied, however. For cereals, participants devoted most attention to sodium (9.1% of time), then fat (6.20%), and calories (6.15%). Participants paid less attention to fiber (5.1%) and vitamin D (1.8%). See Table 2 for means, standard deviations, and p-values. For frozen entrées, participants paid most attention to calories (10.8%, Table 3), followed by fat (9.9%) and fiber (6.7%). Thus, neither of the manipulated nutrients on cereals (i.e., sugar and fiber) received the most attention and only one of the manipulated nutrients on entrées (i.e., calories but not fiber) received much attention.

**Table 2:** Average proportion dwell times by Front-of-Package nutrition types for Cereals. P-values are Tukey-Kramer adjusted for multiple comparisons.

Cereals	Mean Proportion Dwell Time	St. dev.	p-values for all pair-wise comparisons
Calories	0.061	0.027	
Fat	0.062	0.021	
Fiber	0.051	0.025	<0.001
Vitamin D	0.018	0.012	
Sodium	0.091	0.031	
Sugar	0.073	0.028	

**Table 3:** Average proportion dwell times by Front-of-Package nutrition types for entrées. P-values are Tukey-Kramer adjusted for multiple comparisons.

Entrées	Mean Proportion Dwell Time	St. dev.	p-values for all pair-wise comparisons
Calories	0.11	0.035	
Fat	0.099	0.032	<0.001
Fiber	0.067	0.028	

Accuracy was poor for the cereal trials, averaging 38% (SD 28.4%) across participants (Table 1), nearly 12% below and significantly worse than random guessing (P <0.001), implying that many participants made choices based on minor differences in some nutrients while ignoring large difference in others. For frozen entrées, participants were about 13% above chance – about 63% (SD 19.4%) correct – significantly better than cereals, and better than random guessing (P<0.001, for both.)

Although accuracy was low on average, scores ranged from 0–100% across all participants. Hence, we next examined whether 1) attention to specific FOPs was negatively or positively related to accuracy and 2) whether prior knowledge influenced accuracy of healthful decisions, after controlling for attention and for demographic covariates.

For cereals, attention to calories (p-value<0.001), fat (p-value<0.001), and sodium (p-value = 0.025) was negatively associated with accuracy (Table 4). For every percent increase in attention, accuracy declined by 3.7% for calories, 3.1% for fat, and 1.1% for sodium. Attention to fiber and sugar (p-values < 0.001) was positively associated with accuracy. For every percent increase in dwell time, accuracy increased by 4.8% for fiber and 2.9% for sugar. There was no significant association observed for vitamin D (p-value = 0.19).

**Table 4:** Estimated effects of dwell time on accuracy, tested separately for each area of interest (AOI) for cereals.

Cereals	FOP AOI tested in model											
	Calories		Fat		Fiber		Sodium		Sugar		Vitamin D	
Covariate	Est	P	Est	P	Est	P	Est	P	Est	P	Est	P
Proportion Dwell Time on FOP AOI	-3.7	<.001	-3.1	<.001	4.8	<.001	-1.05	.035	2.9	<.001	1.81	.17
Nutrition Knowledge (points)	1.5	.001	1.8	<.001	.90	.036	1.9	<.001	1.2	.014	1.6	.001
Sex (male = 1)	3.2	.3	5.2	.10	.9	.75	4.2	.19	2.3	.44	3.2	.31
Age (years)	-.3	.004	-.10	.14	-.2	.08	-.14	.16	-.2	.04	-.2	.06
Education (years)	.52	.43	.70	.33	0.5	.44	0.64	.36	.5	.48	.68	.33
Income (bracket, see Table 1)	2.0	.009	1.9	.01	1.1	.14	1.9	.02	1.4	.07	1.7	.034
BMI (points)	-.04	0.86	-.08	.74	-0.01	.96	-0.1	.68	.07	.75	.011	.64

Estimates (Est) represent the change in percent accuracy for each unit change in the covariate. Units for the covariates are shown in parentheses. P-values <0.05 were considered statistically significant.

For entrées, only attention to fiber was significantly associated with accuracy. For every percent increase in dwell time on fiber, accuracy increased by 2.4%. Attention to both calories and fat was negatively associated with accuracy, but this association was not statistically significant (p-values = 0.99 and 0.13, respectively). See Table 5.

**Table 5:** Estimated effects of dwell time on accuracy, tested separately for each area of interest (AOI) for entrées, and adjusted for covariates.

Entrées	FOP AOI tested in model					
	Calories		Fat		Fiber	
Covariate	Est	p	Est	p	Est	p
Proportion Dwell Time on FOP AOI	-.25	.99	-.75	.13	2.4	<.001
Nutrition Knowledge (points)	1.1	.001	1.1	<.001	.71	.03
Sex (male = 1)	6.5	.003	6.9	.002	5.29	.01
Age (years)	-.05	.48	-.04	.56	-.04	.53
Education (years)	.23	.63	.24	.61	.17	.72
Income (bracket)	1.2	.03	1.3	.02	.87	.10
BMI (points)	.30	.07	.30	.08	.23	.14

Estimates (Est) represent the change in percent accuracy for each unit change in the covariate. Units for the covariates are shown in parentheses. P-values <0.05 were considered statistically significant.

Nutrition knowledge was strongly associated with accuracy both for cereal packages (all p values ≤ 0.04) and entrée packages (all p values ≤0.03) (Tables 4 and 5). Knowledge was associated with greater accuracy even after adjusting for time spent reading the FOPs, thus the impact of knowledge was not simply to prompt more attention to the FOP.

Demographic variables had some, but not consistent, associations with accuracy (Tables 4 and 5). For cereals, greater accuracy was significantly associated with higher household income (all p-values < 0.05), except in the fiber and sugar models (p-values 0.13 and 0.075, respectively), but not with education (all p-values > 0.2). Accuracy decreased with age in every model except fat, fiber, and sodium (p-values = 0.15, 0.075, and 0.16, respectively). There were no significant differences in accuracy between the sexes (all p-values > 0.1). BMI was not associated with accuracy in any model (all p-values > 0.60). For entrées, sex was strongly associated with accuracy in all models (all p-values less than or equal to 0.012). Women had higher accuracy than men on all models ranging from 5% higher for the fiber model to 7% higher for the fat model. Neither age (all p-values > 0.5) nor education (all p-values > 0.7) was significantly associated with accuracy. Higher BMI was marginally associated with greater accuracy in the fat and calorie models (p-values 0.07 and 0.08, respectively) but not significantly associated in the fiber model (p-value = 0.140).

## Discussion

FOPs have the potential to serve an important role in public health. They could inform individuals about the food products they eat because the labels are prominently placed and offer simplified nutrition information, relative to the more detailed and complex NFPs. However, the data presented here suggest that individuals misunderstand FOPs, which necessarily limits their ability to support healthful dietary choices. Nutrient-specific manipulations, eye tracking methodology, and an assessment of nutrition knowledge were used in the present study to shed light on the processes underlying consumers’ use of FOPs to make food choices based on healthfulness.

Our finding that FOPs can be confusing is consistent with past work investigating a summary FOP system that is not widely used in the US. In this system, foods are rated on a scale based on the number of to-be-limited nutrients that exceed a limit and given a star for each nutrient below that limit [14]. Researchers presented an FOP symbol for a hypothetical cereal (package not presented) to an online panel and found that consumers who saw the 0–3 FOP style were less able to differentiate between more and less healthful FOPs relative to consumers who saw the 1–4 FOP style. This work is important in showing that zeros in this system confuse consumers. Based on past work showing that familiarity supports FOP use [22], findings from the present study add to the literature by showing that even with familiarity provided by the Facts up Front system, individuals are confused when using FOPs to guide healthfulness choices.

The results from the present also add to the literature by showing that FOP comprehension difficulties are not attributable to a failure to consult FOPs. Eye tracking data showed that individuals looked at all nutrients, regardless of whether FOPs had 3 and 6 cells. Thus, although attention to FOPs may help consumers in some cases, attention does not by itself guarantee more healthful product choices. This finding complements recent research showing that consumers pay more attention to FOPs when given healthfulness goals relative to purchase goals [15, 19] as well as research showing that those who consume more healthful diets pay more attention to FOPs during a mock shopping task [23].

Data from the present study also show that some nutrients receive more attention than others when individual select foods based on healthfulness goals. Specifically, calories and fat received relatively more attention than did other nutrients. This is in line with survey data showing that 70% of consumers reported using calorie information on packaged foods, which was a higher percentage than for any other nutrient or ingredient in the survey [1]. Also, 39% of those surveyed in a previous study reported that they often or always select the product with lower total fat when comparing similar products and 57% percent report comparing products on sodium content [24].

Our findings suggest that, although individuals pay attention to calories, fat, and sodium, they may rely on these nutrients to the exclusion of others. That is, differences between two products could be very small on one of these nutrients but still drive a decision without consideration of other important nutrients. We found that the more time individuals spent on calories, fat, and sodium, the lower their accuracy in determining which product was more healthful. As others have noted [11], over-valuing nutrients to limit may lead individuals away from nutrients to encourage such as certain packaged cheeses, for example, which may be high in fat but are rich in other important nutrients such as vitamin D and calcium. The finding that calories received the greatest amount of attention, but failed to consistently lead to the more healthful choice, suggests that proposed changes to Nutrition Facts panels that involve making calorie information more prominent, may not result in more healthful food choices.

We also found that vitamin D was not necessarily over emphasized, but it was confusing. Vitamin D is important because it helps the body absorb calcium, which is important for bone health. Individuals spent more time on vitamin D than they did on fiber, but did not reliably use this information to select the healthier product. On the other hand, attention to fiber and sugar both were associated with greater accuracy, suggesting that those who paid more attention to these nutrients were better able to determine which product was more healthful.

Accuracy was lower in this study (50% on average), relative to some past work [9]. However, we asked individuals to compare two similar products that varied in only one nutrient, whereas Grunert and colleagues presented a somewhat easier task of distinguishing between products that varied in three nutrients: fat, saturated fat and calories. As the results of this study show, individuals allocate a good deal of attention to calories, which may have made the comparisons in the earlier study fairly easy.

The findings surrounding the effects of knowledge on healthfulness choices inform processes underlying the use of FOPs. First, nutrition knowledge was broadly helpful in improving the accuracy of product choices, regardless of personal factors (age, education, sex, or BMI) or FOP factors (3 or 6 cells). Second, the effects of knowledge are present even after controlling for attention, implying that the benefits of knowledge are not limited to getting people to pay more attention to FOPs. In general, these data are inconsistent with the IOM goal that FOPs make minimal demands on processing and avoid reliance on prior nutrition knowledge [2]. Thus, Facts up Front, although familiar to US consumers, may be falling short of that goal. While this study’s findings are compelling, there are several limitations. Although participants were invited to participate based on a stratified cluster sampling, this method does not prevent self-selection. Thus, it is possible that study participants differed from those who declined to participate. Also, we included 24 food choices drawn from two categories of foods. Although this represents more choice than some food label use studies, the results may not generalize to other product types, for example, sugary beverages. In addition, due to the laboratory setting of the study, we cannot draw conclusions about how individuals interpret nutrition labels in the larger context of grocery shopping or selections from menus.

Our findings suggest several recommendations for FOP policy. First, policy makers should carefully consider placing limits on the number of nutrient cells provided on package fronts, since this study and others have shown that the greater number of Facts up Front cells led to more confusion and lower accuracy in selecting a more healthful product. Second, it is critical that FOP labeling include nutrients to encourage (e.g., fiber) to increase a focus on balanced calories and counteract consumers’ focus on calories. Finally, our results suggest that nutrition labels should not increase the size or visibility of calories relative to other nutrients, because calories appear to be over-used and can interfere with selecting a more healthful product.

Roberto and colleagues [13] found that consumers have a generally favorable view of the nutritional value of the foods containing Facts Up Front labels, yet they underestimated amounts of saturated fat and sugar and overestimated amounts of fıber and protein. Misperceptions of nutritional qualities of packaged foods may be even more pronounced in grocery stores in that the Facts Up Front system allows manufacturers to select the nutrients they wish to highlight [13]. Thus, less-healthy products can seem more healthful by virtue of the information provided on the package front (e.g., a product with high saturated fat may not list this nutrient). FOP regulations, informed by rigorous consumer research, are likely to lead to a more effective way to inform consumers and promote healthful food choices [3].
