# Correction: Misunderstanding of Front-Of-Package Nutrition Information on US Food Products

**DOI:** 10.1371/journal.pone.0134772

**Published:** 2015-07-28

**Authors:** The PLOS ONE Staff

## Notice of Republication

This article was republished on June 30, 2015, to replace copyrighted or confidential material. Please download this article again to view the correct version.
